# Macrophage inhibitory factor (MIF) gene polymorphisms are associated with disease susceptibility and with circulating MIF levels in active non‐segmental vitiligo in patients from western Mexico

**DOI:** 10.1002/mgg3.1416

**Published:** 2020-07-23

**Authors:** Alejandra Garcia‐Orozco, Itzel Alejandra Martinez‐Magaña, Annie Riera‐Leal, José Francisco Muñoz‐Valle, Marco Alonso Martinez‐Guzman, Ricardo Quiñones‐Venegas, Gabriela Athziri Sánchez‐Zuno, Mary Fafutis‐Morris

**Affiliations:** ^1^ Centro de Investigación en Inmunología y Dermatología/Instituto Dermatológico de Jalisco “Dr. José Barba Rubio” Centro Universitario de Ciencias de la Salud Universidad de Guadalajara Zapopan Mexico; ^2^ Doctorado en Ciencias Biomédicas con Orientación en Inmunología Departamento de Fisiología Centro Universitario de Ciencias de la Salud Universidad de Guadalajara Guadalajara Mexico; ^3^ Instituto Dermatológico de Jalisco “Dr. José Barba Rubio” Secretaría de Salud Jalisco Zapopan Mexico; ^4^ Instituto de Investigación en Ciencias Biomédicas Centro Universitario de Ciencias de la Salud Universidad de Guadalajara Guadalajara Mexico

**Keywords:** genetic susceptibility, MIF, non‐segmental vitiligo, polymorphisms

## Abstract

**Background:**

The macrophage migration inhibiting factor (MIF) is a protein that promotes the activation of immune cells and the production of other proinflammatory cytokines such as TNF‐α, IL‐1β, and IFN‐γ, which have proposed to play an essential role in the pathogenesis of vitiligo. The study aimed to assess the association between *MIF* polymorphisms (−794 CATT_5‐8_ and −173 G>C), MIF *in situ* expression, and MIF serum concentrations with susceptibility and disease activity in patients with non‐segmental vitiligo (NSV) from western Mexico.

**Methods:**

The study included 111 patients with NSV and 201 control subjects. Genotyping was performed by conventional PCR (−794 CATT_5‐8_) and PCR‐RFLP (−173 G>C) methods. *MIF* mRNA expression was quantified by real‐time PCR and MIF serum concentrations were determined by ELISA kit. Histopathological samples were analyzed by automated immunohistochemistry.

**Results:**

The *MIF* polymorphisms were associated with NSV susceptibility. Serum concentrations of MIF were higher in patients with active NSV and correlated negatively with the years of evolution. The depigmented skin from patients with active vitiligo showed a high expression of MIF.

**Conclusion:**

*MIF* polymorphisms increase the risk of NSV in the western Mexican population. The serum concentrations of MIF and *in situ* expression are associated with active NSV.

## Graphical abstract

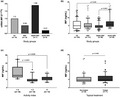

## 1 INTRODUCTION

Vitiligo affects 0.5% to 2% of the world population, placing it as the most common skin depigmenting disorder resulting from a selective loss of epidermal melanocytes (Boniface, Seneschal, Picardo, & Taïeb, 2018; Rodrigues, Ezzedine, Hamzavi, Pandya, & Harris, 2017). Even though it is a complex disease, that combines genetic and environmental factors with metabolic and immune alterations, a significant role of silent inflammation and autoimmunity is demonstrable, in particular, during the progressive phase of the disease (Boniface et al., 2018; Rodrigues et al., 2017; Speeckaert, Speeckaert, De Schepper, & van Geel, 2017).

Depigmentation of the skin and hair follicles is the clinical hallmark of vitiligo, and the extension of the patches allows us to classify the pathology in localized or segmental vitiligo and non‐segmental vitiligo (NSV) (Rodrigues et al., 2017). Vitiligo Global Issues Consensus Conference characterizes non‐segmental vitiligo as an acquired chronic pigmentation disorder with white patches, most often symmetrical increasing in size progressively or during flares with time, corresponding histologically to a substantial loss of functioning epidermal pigment cells and, usually in a second time, of hair follicle melanocytes (Boniface et al., 2018; Ezzedine et al., 2012).

The immunopathogenesis of vitiligo starts with intrinsic abnormalities of melanocytes and keratinocytes, leading up to the activation of the innate immune response and subsequently, the adaptive immunity (Laddha et al., 2013; Picardo et al., 2015). The recruitment of natural killer cells and inflammatory dendritic cells has described when melanocytes from vitiligo patients develop cellular stress and release inflammatory signals (Rashighi & Harris, 2017). Also, an increased number of cytotoxic CD8+ T cells in the blood and epidermis in the affected skin of patients has been seen and the infiltration correlates with the disease severity (Le Poole, van den Wijngaard, Westerhof, & Das, 1996; van den Boorn et al., 2009). Recently, the release of the chemokine ligands CXCL16 by keratinocytes, and CXCL12 and CCL5 by melanocytes in vitiligo under oxidative stress stand out among the physiopathological mechanisms involved in the recruitment of T cells (Li et al., 2017; Rezk et al., 2017).

Another of the pro‐inflammatory cytokines that have recently associated with an increased risk of vitiligo is the Macrophage migration inhibitory factor (MIF) (Farag, Habib, Kamh, Hammam, & Elnaidany, 2018; Ma et al., 2013; Serarslan et al., 2009). It has been shown to play a crucial role in several types of immune and autoimmune diseases. MIF is stored in the cytoplasm in vesicle‐like structures and it is secreted in response to several stimuli including lipopolysaccharide (LPS), tumor necrosis factor (TNF)‐α, hypoxia, hydrogen peroxide (H_2_O_2_), thrombin, and angiotensin II (Jankauskas, Wong, Bucala, Djudjaj, & Boor, 2019). Also, MIF secretion was induced by oxidative stress and DNA damage, both common mediators of a variety of stimulators of MIF secretion (Gupta, Pasupuleti, Du, & Welford, 2016). Also, it is characterized by the fact that it allows the activation of immune cells and the production of proinflammatory cytokines such as TNF‐α, IL‐1β, and IFN‐γ, which have proposed to play an essential role in the pathogenesis of vitiligo (Ma et al., 2013).

Polymorphism in the number of CATT microsatellite repeats at the position −794 of *MIF* promoter affects its transcription (rs5844572). The presence of more than five CATT repeats genotypes is associated with a higher *MIF* promoter activity and has been associated with higher severity of autoimmune diseases (Baugh et al., 2002). SNP −173 G>C (rs755622) located in the promoter region of *MIF* gene (OMIM: 153620) strongly correlates with the severity and susceptibility to several inflammatory and autoimmune pathologies (Castañeda‐Moreno et al., 2018; De la Cruz‐Mosso et al., 2014; Illescas, Gomez‐Verjan, García‐Velázquez, Govezensky, & Rodriguez‐Sosa, 2018; Llamas‐Covarrubias et al., 2013).

The combination of oxidative stress and inflammatory mediators in the pathogenesis of vitiligo and within the physiological mechanisms of MIF release, analyze the polymorphisms that affect the promoter region of the *MIF* gene an important determinant of risk factors for the disease, which may lead to improved prevention and treatment options. That is why in this study, the influence of −794 CATT_5‐8_ and −173 G>C *MIF* polymorphisms in non‐segmental vitiligo and its correlation with serum concentrations, histopathological levels of the protein of patients samples, and disease activity was evaluated.

## 2 MATERIALS AND METHODS

### 2.1 Ethical compliance

The present study was approved by the Ethical Investigation and Biosecurity Committee of the University Center of Health Sciences at the University of Guadalajara (Reference Number CUCS/CINV/0170/17; Guadalajara, Mexico). All research was performed according to the Brazil 2013 amendment of the Declaration of Helsinki (World Medical Association, 2013) and Mexico regulations for studies on human health. Informed consent was signed by all the individuals included in our study.

### 2.2 Subjects

The 312 participants in the study were classified into two groups: 111 patients from the Dermatological Institute of Jalisco “Dr. José Barba Rubio” with a clinical diagnosis of NSV and 201 clinically healthy subjects (CS). None of the CS or their relatives had any evidence of vitiligo. All participants were at least 18 years old of any gender, Mexican mestizo, and native to western Mexico for at least three generations. For the purpose of this study, a Mexican mestizo was defined as a person that was born in Mexico, with the last name of Spanish origin and whose previous three ascending generations were also born in Mexico (Gorodezky et al., 2001). Patients and CS with a body mass index >30, smoking habit, personal history of infectious, immunological, autoimmune/inflammatory or immunosuppressive diseases, pregnant, and lactating women were excluded from the study. None of the patients were having systemic steroid therapy, photo(chemo)therapy, or immunosuppressant treatments.

Patients with NSV were also subclassified according to the vitiligo disease activity (VIDA) score. This score uses a six‐point scale ranging from −1 to +4 based on the presence of new lesions or expansion of existing lesions, where the higher the score represents, the more activity (Ibrahim, Ghaly, El‐Tatawy, Khalil, & El‐Batch, 2014).

### 2.3 Genotyping of −794 CATT_5‐8_ and −173 G>C *MIF* polymorphisms

Genomic DNA was extracted from all subjects from peripheral blood leukocytes by the salting‐out method (Noguera, Tallano, Bragós, & Milani, 2000). The −794 CATT_5–8_ (NC_000022.11:g.23893563_23893564insATTC) *MIF* polymorphism was analyzed by end‐point polymerase chain reaction (PCR) and polyacrylamide gel electrophoresis using the primers reported by Radstake et al. (Radstake et al., 2007). The −173G>C (NC_000022.11:g.23894205G>C) *MIF* polymorphism was genotyped by the PCR‐ restriction fragment length polymorphism (RFLP) technique. Amplification of the polymorphic fragment was performed using the primers reported by Makhija et al. (Makhija, Kingsnorth, & Demaine, 2007); the 366 bp fragment obtained was further digested with the *Alu I* restriction endonuclease (New England Biolabs, Ipswich, MA, USA) by overnight incubation at 37°C. The PCR protocols used in both polymorphisms were as reported by De la Cruz‐Mosso et al. (De la Cruz‐Mosso et al., 2014).

### 2.4 RNA extraction and quantitative real‐time PCR

Total RNA was extracted from 5 ml of peripheral blood of 15 NSV patients and 15 CS, according to Chomczynski–Sacchi technique (Chomczynski & Sacchi, 1987). One microgram of total RNA was converted to cDNA using oligo‐dT and M‐MLV reverse transcriptase (Promega Corp., Madison, WI, USA).

*MIF* mRNA quantification was determined by quantitative real‐time PCR (qPCR) using UPL hydrolysis probes (Roche Applied Science, Germany) and glyceraldehyde 3‐phosphate dehydrogenase (GAPDH) used as a reference housekeeping gene (Cat. No. 05190541001). The PCR reaction was performed on a LightCycler Nano System (Roche Applied Science, Germany). All samples were run in triplicate using the conditions indicated in the UPL Gene Expression Assay protocol in a LightCycler Nano System (Roche Applied Science). After the validation of reaction efficiency, relative expression analysis was performed by the 2^−ΔCq^ and 2^−ΔΔCq^ methods.

### 2.5 Quantification of serum MIF concentrations

Serum was obtained from all individuals at the time of inclusion; cytokine levels were quantified in a subset of 111 NSV patients and 103 control subjects. The determination of serum MIF concentrations was performed by kit LEGEND MAX™ Human Active MIF ELISA (BioLegend®, San Diego, CA, EUA) according to manufacturer's instructions. The MIF assay sensitivity was 17.4 ± 9.2 pg/ml.

### 2.6 Histopathological samples and immunohistochemistry

The histopathological samples were taken from 25 vitiligo patients and 10 control subjects. The samples of the control subjects were obtained from the perilesional area of the lipoma or benign cyst samples from individuals who came to the Institute for their extraction. Additionally, patients with vitiligo were divided into two groups: 15 samples from patients with active disease and 10 patients with stable disease. The biopsies of the patients contained fragments of depigmented skin and perilesional skin in equal parts. Based on the fact that Sutton's nevus shares physiopathological, clinical, and histopathological characteristics with vitiligo, we decided to include five samples from patients with this diagnosis. Written informed consent was signed from all the participants included, to collect and use the tissue specimens for research purposes. After collection, tissues were fixed in 4% formalin and embedded in paraffin.

Serial sections of 4 μm were obtained and fixed in positively charged slides (Cat. No. 6776214; Thermo Scientific). Immunohistochemistry processing and staining were performed using the BenchMark ULTRA automated system (Cat. No. N750‑BMKU‑FS 05342716001; Ventana Medical Systems, Inc, Roche, Tucson, USA). Rabbits and polyclonals primaries antibodies against MIF (Cat. No. sc‐20121 RRID: AB_648587) and CD74 (Cat. No. sc‐20082 RRID: AB_2075501), both from Santa Cruz Biotechnology, Inc., TX, USA; were prepared at a concentration of 1:50 in a 1x concentrated solution of Tris‐buffered saline with Tween 20 (TBS‐T). As a negative control, in some samples, the primary antibody was omitted. The Ventana OptiView DAB IHC detection and OptiView Amplification kit from Ventana Medical Systems, Inc., were used for the staining and amplification of the stain, respectively.

### 2.7 Analysis of the protein expression

Digital image files were obtained at 10× and 40× magnifications using an optical microscope (Carl Zeiss AG, Oberkochen, Germany), coupled with a digital camera CoolSNAP (Photometrics, Tucson, USA). The digital analysis was performed using the cell counter function of the Image‑pro Plus 6.0^®^ software (Media Cybernetics, Inc., Rockville, MD, USA). The optical densities of the brown color were calculated for five fields of each sample.

### 2.8 Statistical analysis

The descriptive analysis, nominal variables were expressed as frequencies; continuous variables with nonparametric distribution were expressed as medians, percentile 10–90, and interquartile ranges 25–75. The genotypic and allelic frequencies of the polymorphisms were performed by direct counting. Hardy–Weinberg equilibrium in control subjects was determined by the chi‐square test (*X* ^2^). The distribution of the genotypes and allele frequencies of the polymorphisms in both groups (NSV patients and control subjects) were analyzed by *X* ^2^ test with 3x2 and 2x2 contingency tables, respectively. To compare nonparametric quantitative determinations, the Mann–Whitney U test, Odds ratio (OR), and 95% confidence interval (95% CI) were used to analyze the risk for NSV associated with the *MIF* gene polymorphisms. To evaluate the effect of both polymorphisms on NSV, we performed dominant inheritance genetic models. For correlation analysis of continuous variables with nonparametric distribution, we used the Spearman correlation test. Obtained data were analyzed with the statistical software SPSS v 23 and GraphPad Prism v 7, considering *p* ≤ 0.05 as statistically significant and 80% statistical power.

## 3 RESULTS

### 3.1 Clinical and demographic characteristics

The clinical and demographic features of the study subjects are summarized in Table 1. The median age of patients was 42 years, and 55% were female. All the participants had a normal weight with a body mass index of less than 25. Approximately 10% of the patients had a family history of vitiligo. Forty‐five patients suffered some degree of disease activity, while the most had a stable state. Most patients (64%) were used topical treatment at the time of the study: 26.2%, medium‐potency corticosteroids, 15.3% a calcineurin inhibitor medication, and 22.5% combination of topical treatment. None of the participants were using systemic therapy. All the patients did not present any other comorbidity at the time of the study and 100% presented negative thyroid function tests (data not shown).

**Table 1:** Clinical and demographic characteristics of NSV patients and CS

Variable	NSV *n* = 111 (%)	CS *n* = 201 (%)
*Sociodemographic characteristics*		
Age (years)^a^	42 (18–82)	38 (18–89)
Gender^b^		
Female	61 (55)	107 (53.2)
Male	50 (45)	94 (46.8)
BMI (kg/m^2^)^a^	22.7 (19.1–23.6)	20.9 (18.7–24.5)
*Clinical characteristics*		
Family history of vitiligo^b^	11 (9.9)	–
Activity^b^		
Active	45 (40.5)	–
Stable	66 (59.5)	
Treatment^b^		
Treated		–
TCs	29 (26.2)	
TCIs	17 (15.3)	
TCs + TCIs	25 (22.5)	
Non‐treated	40 (36)	–

Abbreviations: BMI, body mass index; CS, control subjects; NSV, non‐segmental vitiligo; TCIs, topical calcineurin inhibitors; TCs, topical corticosteroids.

^a^ Data are expressed as median and (p5–p95).

^b^ Data are expressed as the number of individuals and percentage (%).

### 3.2 Association between NSV and *MIF* gene polymorphisms

The genotypic and allelic frequencies of −794 CATT_5‐8_ and −173 G>C *MIF* polymorphisms were determined in patients and CS. No deviation from Hardy–Weinberg equilibrium was observed in both cases (*p *> 0.05). A significant association in the genotype distribution of the −794 CATT_5–8_ *MIF* polymorphism in NSV patients (*p *= 0.02) was found, with an OR of 3.16 (CI = 1.33–7.40; *p* = 0.01) for the 5,7 repeats heterozygote genotype, and an OR value of 1.89 (IC = 1.28–2.79, *p* = 0.001) for the −794 CATT_7_ allele. This evidence indicates that the −794 CATT_7_ allele may increase the risk of NSV in the western Mexican population. According to the dominance model, a statistically significant difference between both groups (*p* = 0.001) and an OR of 2.20 (IC = 1.37–3.53) were found, which suggests that the heterozygous genotypes with allele (‐,7 + 7,7) have 2.20‐fold more susceptibility to present NSV compared with the subjects without the risk allele. Following these observed patterns, we concluded that a single copy of the −794 CATT_7_ allele is enough to increase the risk (Table 2).

**Table 2:** Genotype and allele frequencies of −794 CATT_5‐8_ and −173 G>C *MIF* polymorphisms in NSV patients and CS

	NSV *n* = 111 (%)	CS *n* = 201 (%)	*p* *	OR (CI 95%)	*p* *
	**−794 CATT_5‐8_*MIF***				
**Genotype**			**0.02**		
6,6^a^	30 (27.0)	71 (35.3)		1	
5,5	6 (5.4)	7 (3.5)		2.02 (0.63–6.54)	0.38
5,6	15 (13.5)	53 (26.4)		0.67 (0.33–1.37)	0.35
5,7	16 (14.4)	12 (6.0)		**3.16 (1.33–7.40)**	**0.01**
6,7	34 (30.6)	46 (22.8)		1.75 (0.96–3.23)	0.10
7,7	10 (9.0)	12 (6.0)		1.97 (0.77–5.05)	0.24
**Allele**			**0.002**		
6^a^	109 (49.1)	241 (60.0)		1	
5	43 (19.4)	79 (19.7)		1.20 (0.78–1.86)	0.47
7	70 (31.5)	82 (20.3)		**1.89 (1.28–2.79)**	**0.001**
		**Genetic model**			
**Do**					
‐,‐ ^a^	51 (45.9)	131 (65.2)		1	
‐,7 + 7,7	60 (54.1)	70 (34.8)		**2.20 (1.37–3.53)**	**0.001**
	**−173 G>C *MIF***				
**Genotype**			**0.05**		
GG^a^	46 (41.4)	116 (57.7)		1	
GC	62 (55.9)	75 (37.3)		**2.08 (1.29–3.36)**	**0.003**
CC	3 (2.7)	10 (5.0)		0.76 (0.19–2.87)	0.92
**Allele**					
G^a^	154 (69.4)	307 (76.4)		1	
C	68 (30.6)	95 (23.6)		1.42 (0.99–2.05)	0.07
		**Genetic model**			
**Do**					
GG^a^	46 (41.4)	116 (57.7)		1	
GC + CC	65 (58.6)	85 (42.3)		**1.93 (1.20–3.08)**	**0.008**

Abbreviations: C, cytosine; CI, confidence interval; CS, control subjects; Do, dominant inheritance genetic model; G, guanine; NSV, Non‐Segmental Vitiligo; OR, odds ratio.

^a^ Reference category; (‐,‐ = genotypes without risk allele; ‐,7 = heterozygous genotypes with allele risk). *MIF* GenBank Ref Seq, NC_000022.11.

* Chi‐square test *X* ^2^ considering a level of significance of *p* ≤ 0.05.

Similar to −794 CATT_5‐8,_ the analysis of the genotype distribution evidenced a significant association (*p* = 0.05) with an OR equal to 2.08 (1.29–3.36: *p* = 0.003) of the −173 G>C *MIF* polymorphism with NSV diagnosis. However, for the heterozygote genotype GC, no significant difference in the allelic frequency was found. According to the dominance model, the carriers of the −173*C allele (GC + CC) showed 1.93‐fold risk (OR = 1.93; CI = 1.20–3.08, *p* = 0.008) to develop NSV than carriers of the −173*G allele (GG), indicating that a single copy of C allele is enough to increase the risk (Table 2).

### 3.3 *MIF* mRNA expression and serum MIF concentrations in NSV patients, association with the disease activity

**Figure 1:**
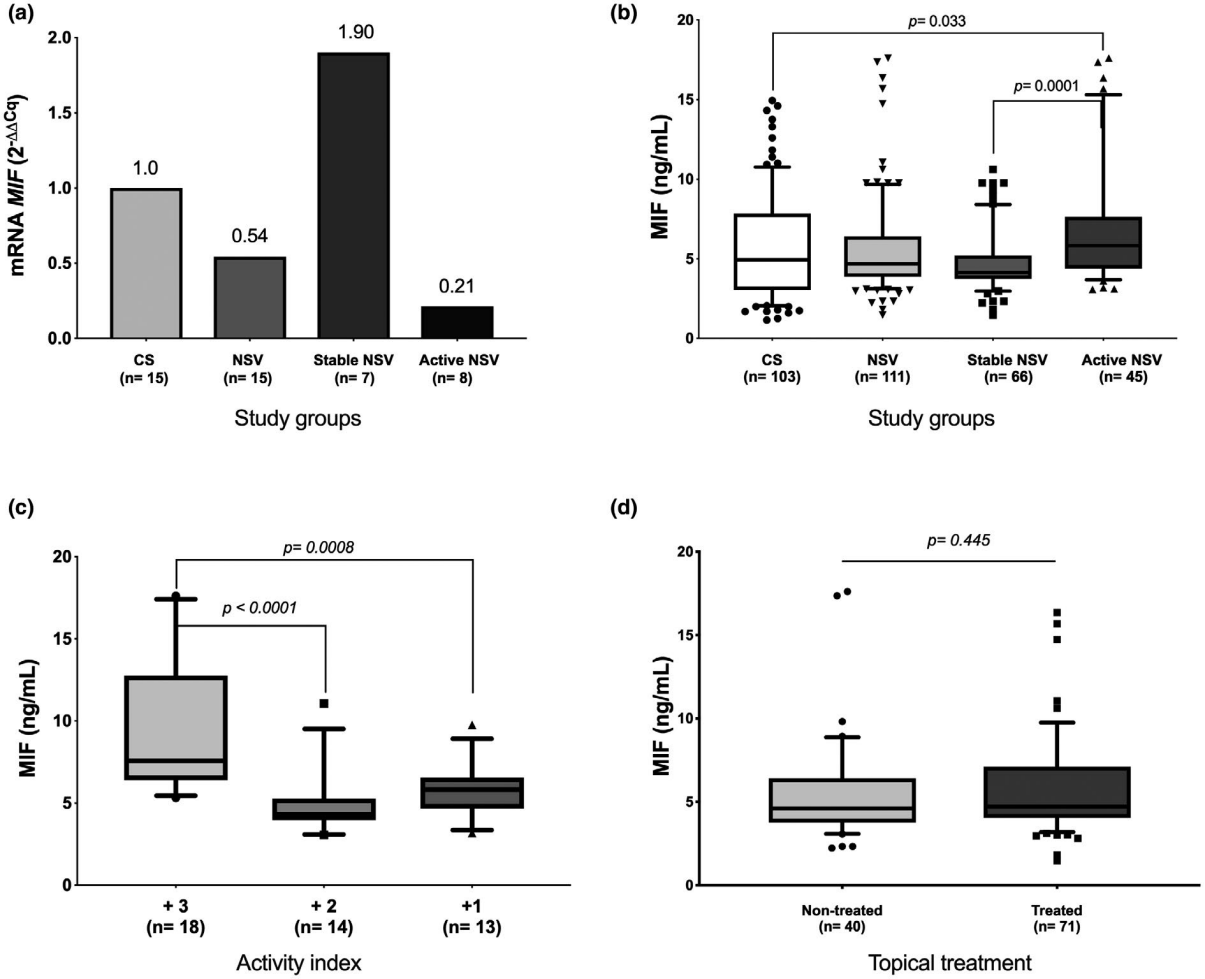
*MIF* mRNA expression and serum MIF concentrations in study groups. (a) Comparison of mRNA expression of *MIF* in study groups; (b) serum MIF concentrations in study groups; (c) serum MIF concentrations of active patients according to the activity index; (d) serum MIF concentrations in NSV patients according to the treatment. Relative *MIF* gene expression was determined by the 2^−ΔΔCq^ method using GAPDH as a reference gene. The MIF concentrations were expressed in medians (p25‐p75); *p*‐value: Mann–Whitney U test. NSV: non‐segmental vitiligo, CS: control subjects, MIF: macrophage migration inhibitory factor

The relative mRNA expression of *MIF* was evaluated in all groups (Figure 1a). The analysis by the 2^−ΔΔCq^ method showed that *MIF* mRNA expression in NSV patients and active NSV was 0.54‐ and 0.21‐fold, respectively, less compared to *MIF* mRNA expression in CS. Moreover, the *MIF* mRNA expression in stable NSV was 1.90‐fold higher than the CS. When these data were analyzed by the 2^−ΔCq^ method, these differences were not statistically significant (*p* = 0.246, data not shown).

We determined if there were differences in serum MIF concentrations between patients and CS. The comparison of the levels between both groups was not significant. Nevertheless, when we stratified the serum MIF concentrations according to the activity index of the patients, a higher MIF concentration was found in active patients in comparison with the stable ones [5.83 ng/ml (4.39–7.64) vs. 4.16 ng/ml (3.75–5.35); *p* = 0.0001]; and with the CS [5.83 ng/ml (4.39–7.64) vs. 4.94 ng/ml (3.05–7.84); *p* = 0.033] (Figure 1b). Moreover, patients with active vitiligo and a score of the activity index equal to or greater (≥) than +3 had higher concentrations of MIF [p <0.0001 compared to +2 punctuation and *p* = 0.0008 compared to +1 score]; (Figure 1c).

To evaluate if the use of topical treatment influenced the serum MIF concentrations and the interpretation of our results, we compared the mean values of both groups. Any significant difference was found (*p* = 0.445) (Figure 1d).

### 3.4 Circulating levels of MIF correlated with the time of evolution and activity of the disease

**Figure 2:**
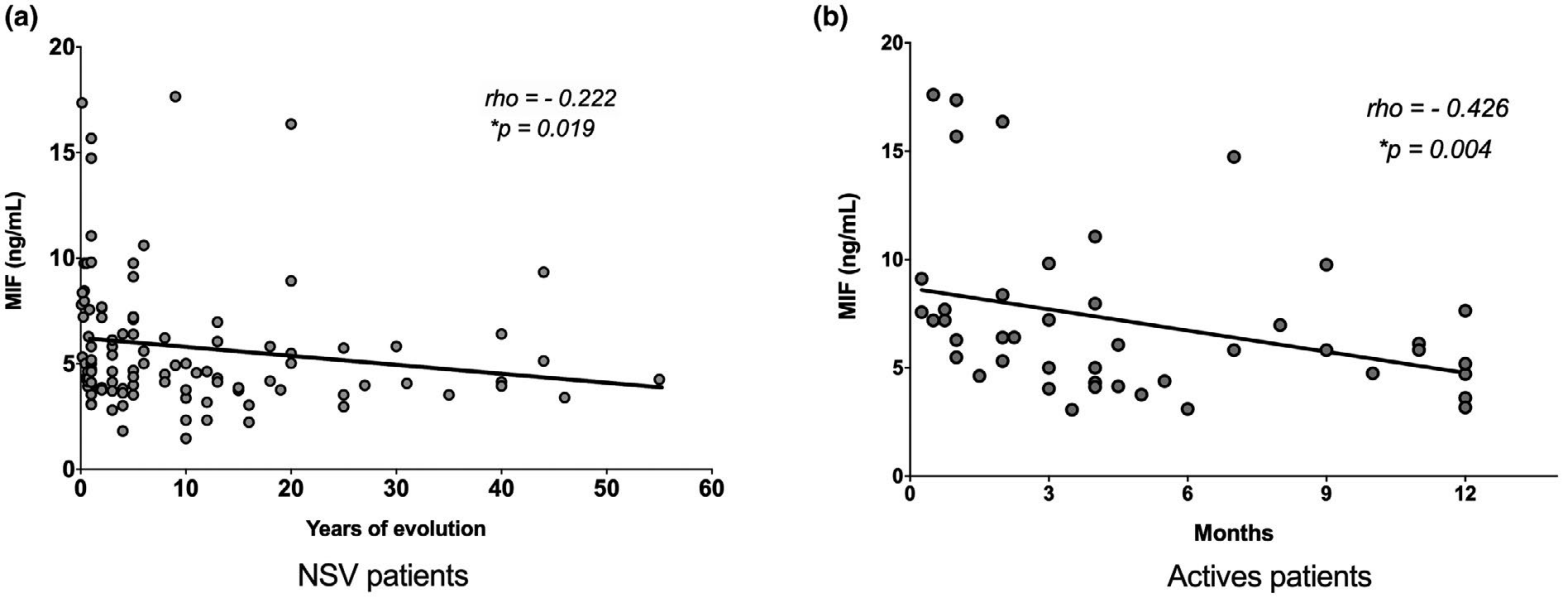
Correlation of MIF and the disease evolution. (a) Serum MIF concentrations negatively correlated with the years of disease evolution (*n* = 111); (b) serum MIF concentrations negatively correlated with the disease evolution in active NSV patients (*n* = 45). *p*‐value: Rho Spearman test

Correlation analysis revealed that the MIF serum concentrations correlated negatively with the years of evolution (*r* = −0.222, *p* = 0.019; Figure 2a) and with the activity of the disease (*r* = −0.426, *p* = 0.004; Figure 2b).

### 3.5 High circulating levels of MIF were associated with *MIF*‐polymorphisms in active vitiligo

**Figure 3:**
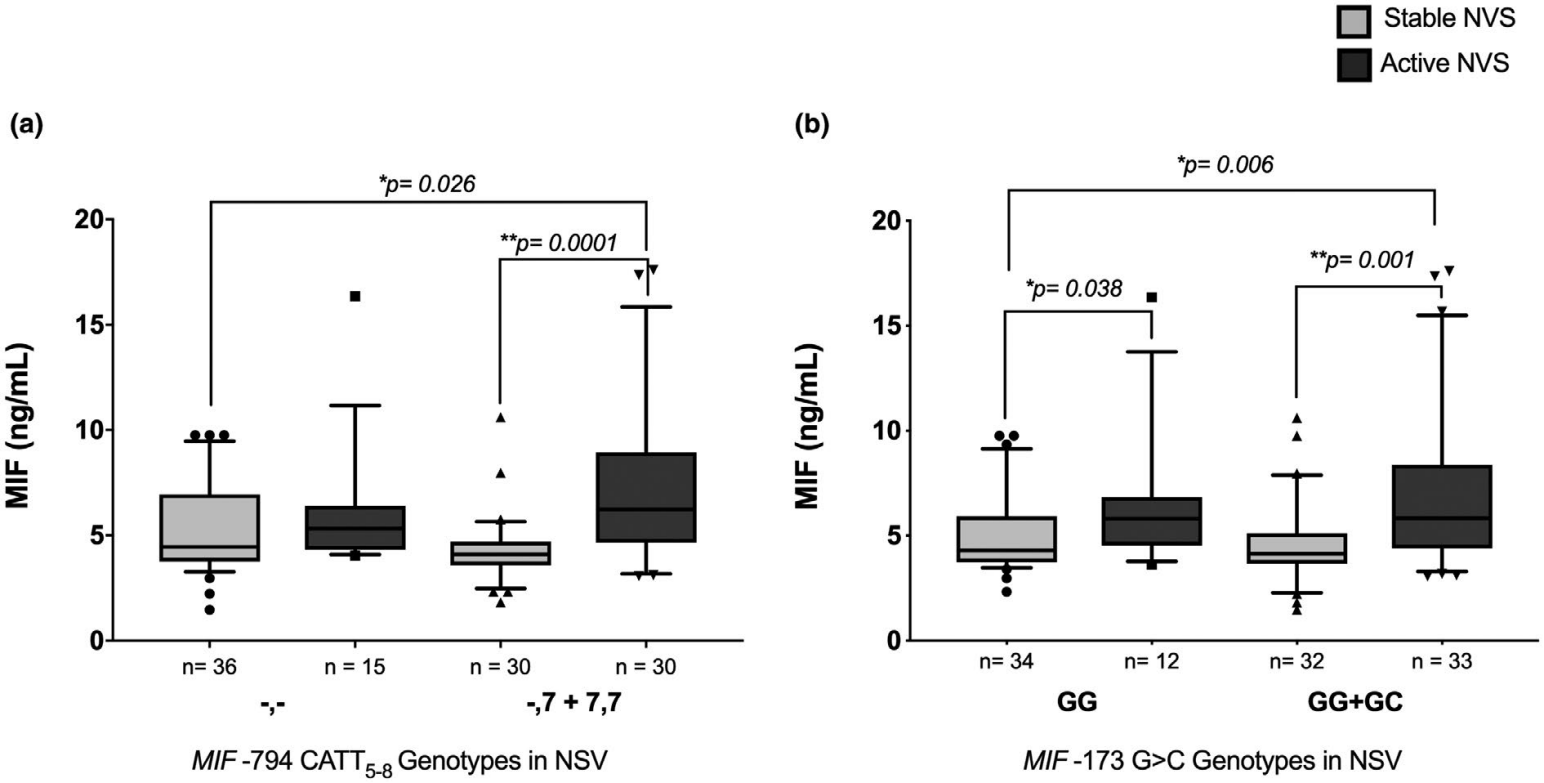
Serum MIF concentrations according to the genetic model of dominant inheritance. The concentrations of MIF in patients with NSV were compared according to the (a) genetic model of dominant inheritance by STR −794 CATT_5‐8_ *MIF* genotypes and (b) genetic model of dominant inheritance by SNP −173 G>C *MIF* genotypes. Data were expressed in medians (p25‐p75); *p*‐value: Mann–Whitney U test. NSV: non‐segmental vitiligo, MIF: macrophage migration inhibitory factor, (‐,‐) = genotypes without risk allele, (‐,7) = heterozygous genotypes with allele risk. *MIF* GenBank Ref Seq, NC_000022.11

The MIF serum concentration in patients was compared by genotypes grouped according to the dominance model proposed for each polymorphism (Figure 3). For −794 CATT_5–8_ *MIF* polymorphism, active NSV patients' carriers of genotypes with the −794 CATT_7_ allele risk showed a significant increase in MIF serum concentrations compared with the stable NSV patients carriers of genotypes without the allele risk (‐,‐) (6.23 vs. 4.45 ng/ml; *p* = 0.026) and with the stable NSV patients carriers of genotypes with −794 CATT_7_ allele risk (6.23 vs. 4.10 ng/ml; *p* = 0.0001) (Figure 3a).

The analysis of −173 G>C *MIF* polymorphism revealed that stables NSV patients with GG genotype had 4.29 ng/ml of MIF, while actives who are carrying genotypes with the −173*C risk allele (GC + CC) had an increase of 5.81 ng/ml; this difference was statistically significant (*p* = 0.038). Moreover, active NSV patients carrying the genotypes GC and CC showed a significant increase in MIF levels (5.83 ng/ml vs. 4.29 ng/ml; *p* = 0.006) and active NSV patients carriers of GG genotype (4.14 ng/ml; *p* = 0.001) (Figure 3b).

### 3.6 *In situ* involvement of MIF

**Figure 4:**
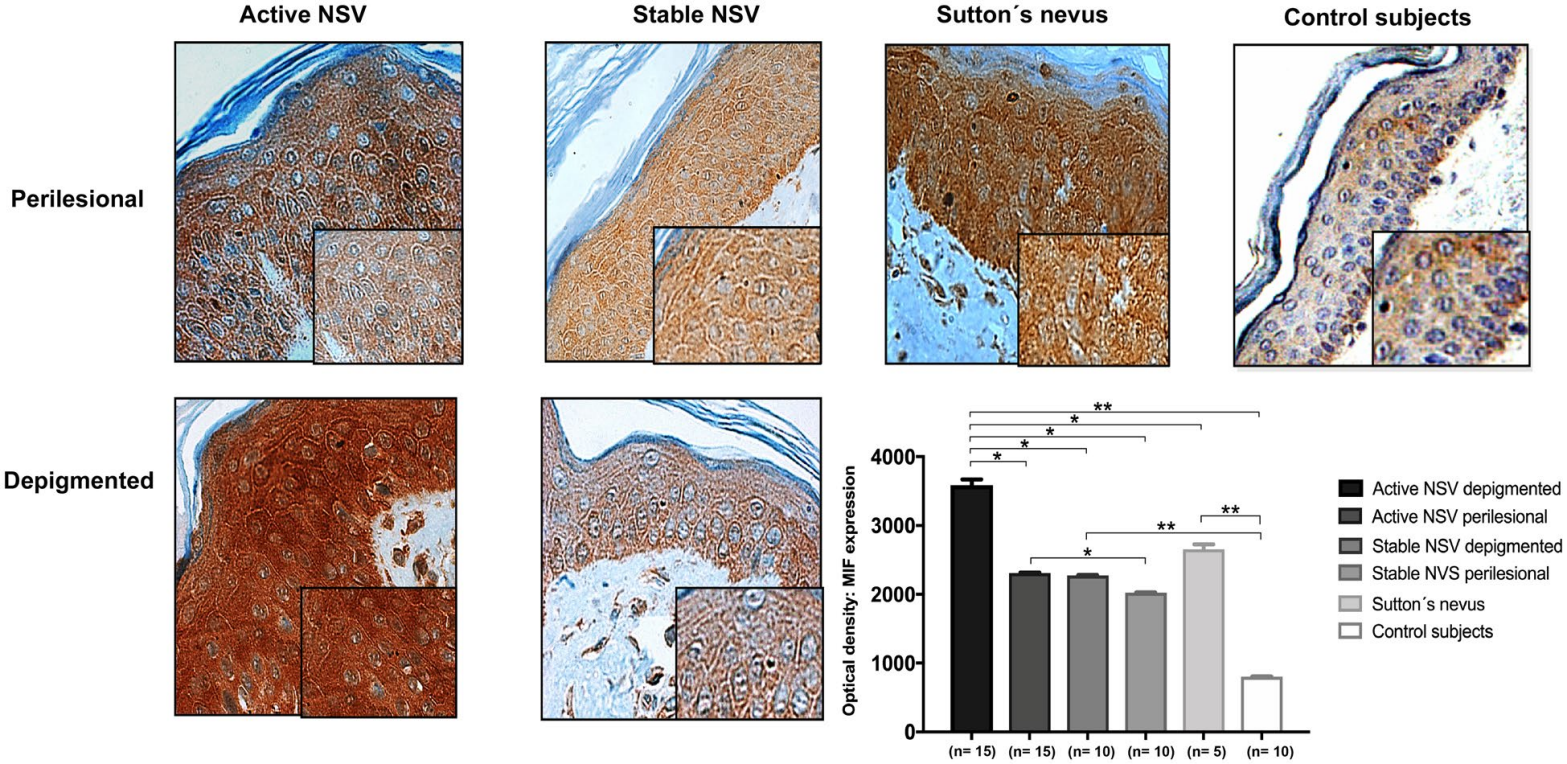
*In situ* expression of MIF was detected in active NSV, stable NSV, Sutton´s nevus, and control subjects using immunohistochemistry; brown coloration indicated positive staining. Images shown are representative of 10 fields (magnification, ×10; magnification inset images, ×40). The small boxes represent the magnified regions. (*p < 0.05; ** *p < *0.01) *p*‐value: Mann–Whitney U test. NSV: non‐segmental vitiligo, MIF: macrophage migration inhibitory factor

The depigmented skin from patients with active vitiligo showed a high expression of MIF, which was significantly higher than the appearance of the protein in the rest of the groups (*p* < 0.05 in all cases). Also, perilesional skin samples of active patients showed significantly higher levels of MIF compared to the perilesional skin of stable patients (*p* = 0.04). Both the depigmented skin and perilesional skin of the 25 patients with vitiligo had higher values of MIF expression compared to the skin sample of control subjects (*p* < 0.01). The Sutton nevus samples had high MIF levels compared to control subjects (*p* = 0.002); however, the average optical densities were significantly lower compared to the samples from the active vitiligo lesions (*p* = 0.03) (Figure 4).

## 4 DISCUSSION

Vitiligo is a skin disease mediated by autoreactive CD8^+^T cells that destroy melanocytes, the pigment‐producing cells, resulting in disfiguring depigmented macules and patches. Even though the precise etiology of vitiligo remains obscure and many factors have been implicated in the development of the disease, the involvement of the immune system is a decisive factor. Macrophage migration inhibitory factor (MIF) is known to participate in immune‐mediated diseases, including rheumatoid arthritis, systemic lupus erythematosus, and some autoimmune skin diseases. However, its role in the pathophysiology of vitiligo has been little explored. Although there are at least 200 genetic polymorphisms described within the MIF gene (Donn et al., 2002), only two identified in the promoter region appear to have functional importance: the −794 CATT_5‐8_ and −173 G>C. Both have been associated with increased serum MIF circulation levels in different populations.

This is the first study that describes the presence of the −794 CATT_5‐8_ and the −173 G>C polymorphisms associated with an increased risk of presenting non‐segmental vitiligo. Previous studies have determined the frequency of the *MIF* promoter polymorphisms and their contribution for systemic autoimmune diseases such as rheumatoid and juvenile idiopathic arthritis (Donn et al., 2004; Llamas‐Covarrubias et al., 2013), systemic lupus erythematosus (De la Cruz‐Mosso et al., 2014; Sreih et al., 2011), sarcoidosis, and multiple sclerosis (Castañeda‐Moreno et al., 2018); or infectious pathologies such as active pulmonary tuberculosis (Kuai et al., 2016) and malaria (Awandare, Martinson, Were, Ouma, & Gregory, 2009). There are reported associations between high‐expression MIF alleles and improved outcomes in pneumonia and meningococcal meningitis (Kuai et al., 2016; Renner et al., 2012). Besides, the main impact of high‐expression of MIF alleles on the severity of the clinical phenotype in asthma (Mizue et al., 2005), systemic sclerosis (Castañeda‐Moreno et al., 2018), and inflammatory bowel disease (Nohara et al., 2004), have been described.

Despite several previous studies that reported an increased risk and severity of inflammatory diseases and reduced response to glucocorticoid medication (Barton et al., 2003); the genetic contribution of *MIF* promoter polymorphisms to some pathologies susceptibility and phenotype is unclear. To clarify, as definitively as possible, the contribution of *MIF* promoter polymorphisms to vitiligo risk and phenotype in this study, we also determined the relationship between −794 CATT_5‐8_ and −173 G>C polymorphisms and the serum levels of MIF. In the first instance, there were no significant differences between patients and controls in terms of serum MIF levels. Moreover, serum MIF concentrations were not related to the presence of polymorphisms in patients with vitiligo.

MIF has been named as “an incriminating agent in dermatological disorders” (Pazyar, Feily, & Yaghoobi, 2013). However, few studies analyze its role in the pathophysiology of dermatological diseases. In vitiligo, this statement is based only on the results of two independent studies. Serarslan and cols. Evaluated the serum of 30 patients and 30 control subjects. The authors concluded that MIF would have an important role in vitiligo since the mean serum MIF level of patients was higher than that of controls.

Interestingly, the author found a significant difference between patients with generalized vitiligo and those who had the localized variant, still, there was no correlation between MIF levels and the disease activity (Serarslan et al., 2009). The other study conducted by Ma et al. analyzed the serum MIF concentrations and mRNA levels in PBMCs of 44 vitiligo vulgaris patients and 32 controls. Both MIF serum levels and mRNA were significantly higher in PBMCs from patients than controls. Also, there was a significant difference between progressive and stable patients, and the vitiligo area severity index score (VASI) of patients correlated positively with changes in both serum MIF concentrations and mRNA levels (Ma et al., 2013). More recently, Farag carried out a study with 50 patients with different degrees of vitiligo severity and 15 healthy controls. Serum MIF concentrations and MIF mRNA levels were significantly highly elevated in patients with vitiligo vulgaris compared to controls, in generalized vitiligo compared to the localized one, and it was a positive correlation with the vitiligo type, duration, and severity (Farag et al., 2018).

The previously exposed evidence presents severe limitations to be able to define the role of MIF in vitiligo. At first glance, all three studies analyzed a small sample of patients. The selection criteria of the patients were not uniform among the three studies and factors that can modify MIF concentrations such as body mass index and cigarette smoke were not considered. The role of MIF in human adipose tissue was first explained by Skurk et al., who demonstrated that adipocytes, as well as preadipocytes, released significant amounts of MIF, and the protein could be localized in the cytoplasm of both the cell types (Skurk et al., 2005). Later, the stronger association of MIF in obese individuals and the relationship with some specifics MIF genotypes have been evidenced by other authors (Kim, Pallua, Bernhagen, & Bucala, 2015; Nishihira & Sakaue, 2012; Sakaue et al., 2006). The capacity of cigarette smoke to alter MIF expression and the susceptibility to suffer from human chronic lung diseases has been proven, and in the murine model, chronic cigarette smoke exposure resulted in decreased *MIF* mRNA and protein expression in the intact lung (Fallica et al., 2014, 2016). Other deficiencies in published studies about MIF and vitiligo include: Differences in the measurement of vitiligo lesions by different evaluators, different criteria to evaluate the activity index and the progressive or stable character of vitiligo, and only one study defined *MIF* mRNA levels directly in a minimal number of histopathological skin samples of patients (Ma et al., 2013).

Then, we decided to stratify the patients according to the activity index of vitiligo. Similar to Ma and Farag, a significant difference in MIF concentrations between the active vitiligo patients compared to the stable ones wAS found. Even more significant, the degree of activity of vitiligo showed a correlation with the presence of −794 CATT_5‐8_ and the −173 G>C polymorphisms and the serum concentrations of MIF. These data suggested that even when there is a genetic risk to suffer the disease, MIF has dynamic changes during different disease status. Eating habits, lifestyle, and other epidemiological factors could intervene in the analysis of MIF participation in the pathophysiology of diseases. Even when evaluating the evolution of the activity of the disease in months, we found that patients with more significant activity and shorter “active” progression have higher levels of serum MIF. These results have also been observed in other autoimmune diseases by our research group (Llamas‐Covarrubias et al., 2012, 2013). A possible explanation for this is that MIF is involved in the early stages of the disease‐promoting proinflammatory synthesis. The prognostic utility for MIF in predicting acute states of different pathologies has been described (Grieb, Merk, Bernhagen, & Bucala, 2010). It has been proposed as a biomarker in central nervous system infection (Østergaard & Benfield, 2009), acute pancreatitis and pancreatic necrosis (Rahman, Menon, Holmfield, McMahon, & Guillou, 2007), acute pyelonephritis (Otukesh et al., 2009), and different causes of severe sepsis (Østergaard & Benfield, 2009) Thus, MIF appeared to be a biomarker for acute pathologies such as active vitiligo and critical illness.

Since its discovery, MIF has been assumed an important role as a pro‐inflammatory cytokine; however, at present, MIF is also believed to control the inflammatory “set point” by regulating the release of other pro‐inflammatory mediators (Kuai et al., 2016). MIF secretion was induced rather than inhibited by glucocorticoid hormones, and this system controls inflammatory and immune responses (Calandra et al., 1995; Calandra & Roger, 2003). MIF normally circulates in plasma, and its levels rise together with adrenocorticotropic hormone in response to stress or invasive stimuli, the hormone stimulates adrenal glucocorticoid production (Flaster, Calandra, & Bucala, 2007). Also, MIF is expressed constitutively by several cell types, including the epithelial lining of tissues in direct contact with the external environment, positioning MIF as a regulator of host responses (Calandra & Roger, 2003). It is considered that it could have a fundamental role in the differentiation of the normal epithelium in the skin (Shimizu, Ohkawara, Nishihira, & Sakamoto, 1996). This evidence and our results open new avenues about the possible role of MIF in vitiligo. Molecular studies are required to evaluate whether MIF has a protective role in patients with active vitiligo and short evolution trying to regulate the inflammatory response or if MIF is a part of the courtship of pro‐inflammatory cytokines that cause the clinical manifestations.

Since most of our patients were receiving treatment, we wanted to evaluate if this variable affected the interpretation of our results by modifying the MIF concentrations. There were no differences in the serum concentrations of MIF between patients with vitiligo in treatment and without treatment. Commonly used repigmentation therapies for vitiligo that are supported by data from randomized controlled trials include topical agents such as corticosteroids and calcineurin inhibitors (Boniface et al., 2018). In the last decades, corticosteroid derivatives have been designed to simultaneously improve efficacy without reaching the systemic circulation to avoid systemic adverse effects (Gual, Pau‐Charles, & Abeck, 2015). Esterification, increase lipophilicity, and improvement of the characteristics of the vehicle, together with the proper indication and use, have been reported to enhance potency, while improving the safety profile of the molecule (Chi et al., 2017; Gual et al., 2015). Another treatment frequently used are calcineurin inhibitors (Cavalié et al., 2015). They offered alternative to topical corticosteroids, since for the treatment of some diseases, like vitiligo, they are as effective or more effective than mild topical corticosteroids with fewer adverse effects (Carr, 2013; Sisti, Sisti, & Oranges, 2016).

It should be noted that the global values of MIF in both study groups were not found above the standard reference values reported in serum (2–6 ng/ml) (Petrovsky et al., 2003). This could indicate a possible *in situ* and non‐systemic involvement of MIF. That is why, in this study, we also evaluate the presence of MIF protein in histopathological samples of patients with vitiligo and compare them with the expression in healthy tissues. Significantly higher levels of MIF protein were found in the samples from patients with vitiligo, especially in the depigmented skin of patients with disease activity. According to our results, Ma et al., 2013 found that MIF mRNA levels were significantly higher in lesional than in healthy skin (Ma et al., 2013). A limitation of this study is that the analysis of mRNA was only carried out in 15 patients and 15 subjects, so new studies are required to ascertain our results. In conclusion, the *MIF* gene polymorphisms increase the risk of NSV in the western Mexican population. However, further studies with a large sample are necessary to verify the association between NSV and the *MIF* gene polymorphisms. Moreover, the serum concentrations of MIF and *in situ* expression are associated with active NSV.

## CONFLICTS OF INTEREST

No conflict of interest was declared by the authors.

## AUTHOR CONTRIBUTIONS

Alejandra Garcia‐Orozco performed the genotyping, statistical analysis, and drafted the manuscript. Itzel Alejandra Martinez‐Magaña and Ricardo Quiñones‐Venegas diagnosed and treated the patients. Annie Riera‐Leal participated in sample collection, sample processing, and provided immunohistochemical assistance. Gabriela Athziri Sánchez‐Zuno performed mRNA extraction and real‐time PCR experiments. Marco Alonso Martinez‐Guzman participated in the interpretation of results and statistical analysis of data. Mary Fafutis‐Morris and José Francisco Muñoz‐Valle designed the study, co‐supervised the work, and revised the manuscript. All the authors have read and approved the final manuscript.
